# Supplementary material for: Developmental increases in white matter network controllability support a growing diversity of brain dynamics
Source: Nat Commun. 2017 Nov 1;8:1252. doi: 10.1038/s41467-017-01254-4 (PMC5665937; doi:10.1038/s41467-017-01254-4)
Supplement: Supplementary file 1 — Supplementary Information [file 41467_2017_1254_MOESM1_ESM.pdf]

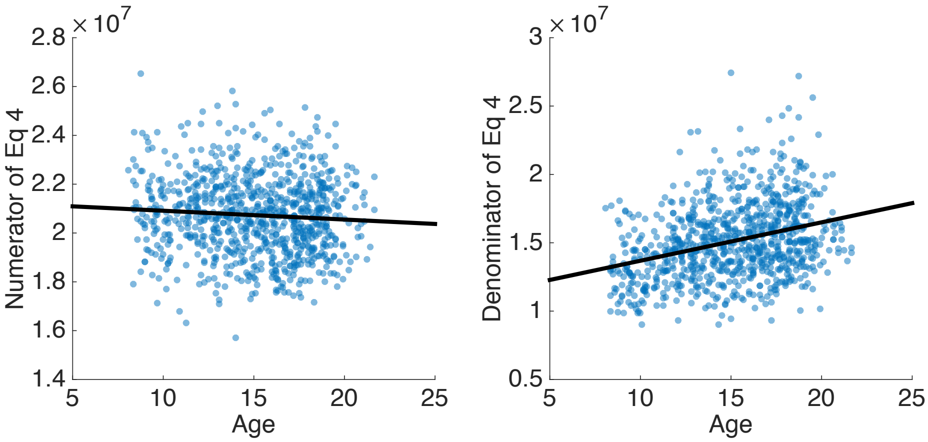

Supplementary Figure 1: **Numerator and denominator of synchronizability with age in years.** (Left) The numerator of the synchronizability function: d^2, where $d := \frac{1}{N}\sum_i \sum_{j\neq i} A_{ij}$ is the average coupling strength per node. (An overall factor of $N-1 = 233$ is the same for all subjects and hence is left out). (Right) The denominator of the synchronizability function $\sum_{i=1}^{N-1} |\lambda_i - \bar{\lambda}|^2$, where $\bar{\lambda} := \frac{1}{N-1}\sum_{i=1}^{N-1} \lambda_i$. We see that the denominator varies much more with age than the numerator, and hence synchronizability is driven by variation in the eigenspectrum (denominator) much more than by differences in connection density (numerator).

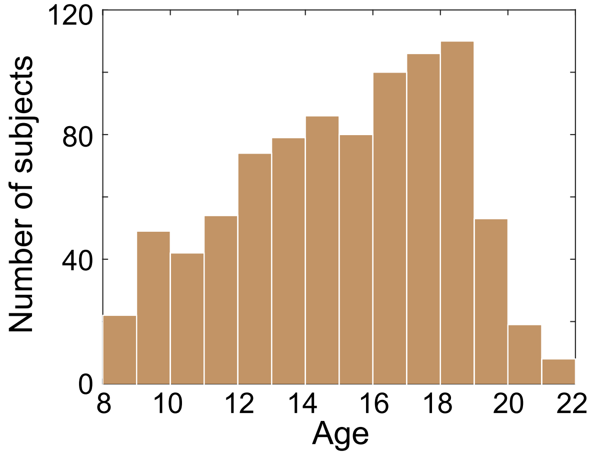

Supplementary Figure 2: **Histogram of subject ages.** We examine 882 healthy individuals from the ages of 8 to 22.

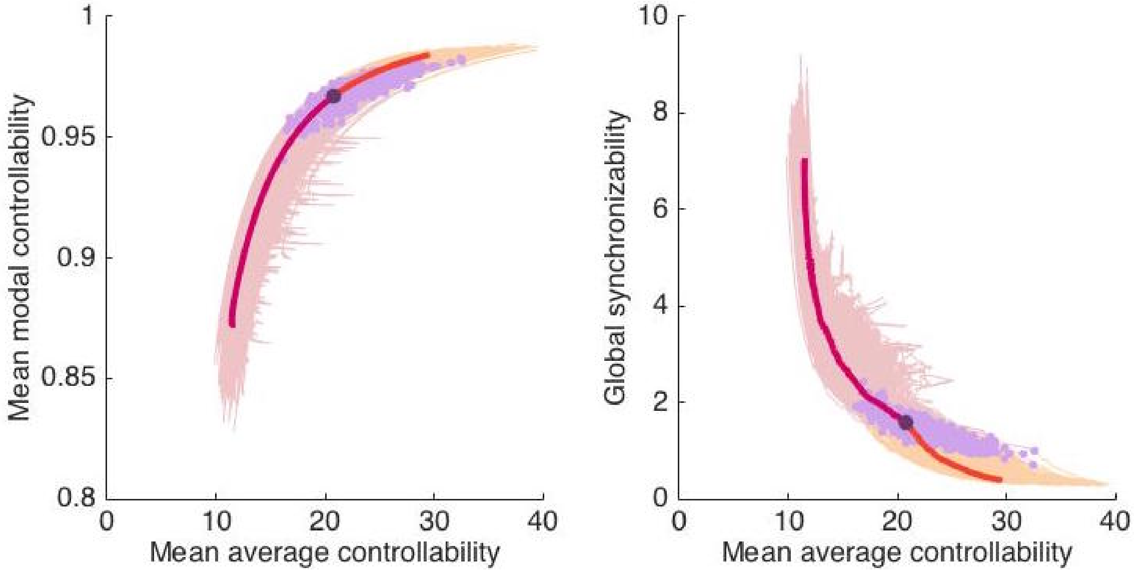

Supplementary Figure 3: **Trajectories without truncation.** While Pareto optimization trajectories in the forward direction (yellow) display very smooth curves, trajectories in the backwards direction (pink) do not. These trajectories were truncated where the curve gradient in the controllability plane (left plot) became negative, often resulting in little loss of overall trajectory length.

Supplementary Table 1: **Average super-controllers.** List of brain regions that strongly increase in average controllability with age, with their laterality and whether they are found in the cortex or subcortex. Labels are standard designations from the 125-scale Lausanne atlas.

**Average super-controllers**		
Region	Hemisphere	Cortex/subcortex
Superior frontal 2-3	Right	Cortex
Precentral 6	Right	Cortex
Paracentral 3	Right	Cortex
Superior parietal 3 & 5	Right	Cortex
Superior frontal 8	Left	Cortex
Precentral 13	Left	Cortex
Paracentral 2	Left	Cortex
Postcentral 1	Left	Cortex
Superior parietal 1 & 5	Left	Cortex
Precuneus 1 & 3-4	Left	Cortex
Brainstem	Left	Cortex

Supplementary Table 2: **Modal super-controllers.** List of brain regions that strongly increase in modal controllability with age, with their laterality and whether they are found in the cortex or subcortex. Labels are standard designations from the 125-scale Lausanne atlas.

**Modal super-controllers**		
Region	Hemisphere	Cortex/subcortex
Lateral orbito frontal 1-4	Right	Cortex
Pars orbitalis 1	Right	Cortex
Medial orbito frontal 2	Right	Cortex
Pars triangularis 1-2	Right	Cortex
Pars opercularis 1-2	Right	Cortex
Rostral middle frontal 1-4 & 6	Right	Cortex
Caudal middle frontal 1-3	Right	Cortex
Precentral 4	Right	Cortex
Paracentral 1-3	Right	Cortex
Rostral anterior cingulate 1	Right	Cortex
Caudal anterior cingulate 1	Right	Cortex
Posterior cingulate 1-2	Right	Cortex
Isthmus cingulate 1	Right	Cortex
Postcentral 1-4	Right	Cortex
Supramarginal 1-4	Right	Cortex
Superior parietal 1-6	Right	Cortex
Inferior parietal 2 & 5	Right	Cortex
Precuneus 3	Right	Cortex
Cuneus 1	Right	Cortex
Lateral occipital 4 -5	Right	Cortex
Lingual 2-3	Right	Cortex
Fusiform 1-2 & 4	Right	Cortex
Parahippocampal 1	Right	Cortex
Entorhinal 1	Right	Cortex
Inferior temporal 1 & 4	Right	Cortex
Middle temporal 1-4	Right	Cortex
Bankssts 1	Right	Cortex
Superior temporal 1-5	Right	Cortex
Transverse temporal 1	Right	Cortex
Insula 1-3	Right	Cortex
Putamen	Right	Subcortex
Pallidum	Right	Subcortex
Hyppocampus	Right	Subcortex
Amygdala	Right	Subcortex
Lateral orbito frontal 2-3	Left	Cortex
Pars orbitalis 1	Left	Cortex
Pars triangularis 1	Left	Cortex
Pars opercularis 2	Left	Cortex
Rostral middle frontal 1-6	Left	Cortex
Superior frontal 2, 4 & 6	Left	Cortex
Caudal middle frontal 2-3	Left	Cortex
Precentral 3 & 6	Left	Cortex
Rostral anterior cingulate 1	Left	Cortex
Caudal anterior cingulate 1	Left	Cortex
Posterior cingulate 1-2	Left	Cortex
Isthmus cingulate 1	Left	Cortex
Postcentral 2-7	Left	Cortex
Supramarginal 1-2 & 4-5	Left	Cortex
Superior parietal 2-3 & 6	Left	Cortex
Inferior parietal 2-3 & 5	Left	Cortex
Precuneus 5	Left	Cortex
Lateral occipital 4	Left	Cortex
Lingual 1-4	Left	Cortex
Fusiform 1-4	Left	Cortex
Parahippocampal 1	Left	Cortex
Entorhinal 1	Left	Cortex
Temporal pole 1	Left	Cortex
Inferior temporal 1-2 & 4	Left	Cortex
Middle temporal 1 & 3-4	Left	Cortex
Bankssts 1-2	Left	Cortex
Superior temporal 1-5	Left	Cortex
Transverse temporal 1	Left	Cortex
Insula 1-2 & 4	Left	Cortex
Caudate	Left	Subcortex
Putamen	Left	Subcortex
Pallidum	Left	Subcortex
Hyppocampus	Left	Subcortex

SUPPLEMENTARY RESULTS: REPLICATION OF RESULTS

Adult subsample

In order to separate our findings on individual variability displayed in controllability and synchronizability and verify that they hold independent of developmental effects, here we examine only the 190 subjects aged 18 and older. By repeating the analyses just on this subject group, we can verify if they already replicate the relationships shown in controllability and synchronizability metrics.

Adults whose brains display high average controllability also tend to display high modal controllability: Pearson's correlation coefficient $r = 0.85$, $df = 189$, $p < 1 \times 10^{-5}$. Further, the brain networks that are more synchronizable tend to display lower average controllability ($r = -0.84$, $df = 189$, $p < 1 \times 10^{-5}$;) as well as lower modal controllability ($r = -0.78$, $df = 189$, $p < 1 \times 10^{-5}$). Hence, this smaller adult sample demonstrates well these relationships between controllability and synchronizability, independent of age-related effects. In subsequent sections below, we continue to first examine this smaller adult sample as well as developmental trends across the entire youth sample.

Controlling for network strength

As networks with higher strength (weighted degree, or sum of all edge weights) tend to have higher average controllability values, here we verify that network strength is not a confound in our results. We do this by first dividing each network by its average strength, to ensure that each matrix has the same average strength of 1 — then repeat our analysis. We find that the 190 adults show an even stronger correlation between mean average controllability and mean modal controllability: Pearson's correlation coefficient $r = 0.89$, $df = 189$, $p < 1 \times 10^{-5}$. These controllability metrics continue to display strong negative correlations with synchronizability: $r = -0.85$, $df = 189$, $p < 1 \times 10^{-5}$ for mean average controllability and $r = -0.78$, $df = 189$, $p < 1 \times 10^{-5}$ for mean modal controllability respectively.

In the larger youth sample from ages 8 to 22, the relationships between controllability metrics and age also persist. Mean average controllability remains positively correlated with age ($r = 0.32$, $df = 881$, $p < 1 \times 10^{-5}$) and mean modal controllability does as well ($r = 0.22$, $df = 881$, $p < 1 \times 10^{-5}$). As synchronizability is calculated independent of the matrix normalization, that result remains unchanged. All these results control for sex, brain volume, handedness and head motion. The emergence of 'super-controllers' – regions of higher average and modal controllability that increase more with age, is also still present. Regions that display increasing average controllability with age are positively correlated with their average controllability values: $r = 0.50$, $df = 233$, $p < 1 \times 10^{-5}$, just as regions that display increasing modal controllability with age are also positively correlated with their modal controllability values: $r = 0.37$, $df = 233$, $p < 1 \times 10^{-5}$. Lastly, the stable controllers in the subcortical region still show a negative correlation between the mean average controllability in those regions, and the subject's IQ (Spearman correlation coefficient $\rho = -0.14$, $df = 881$, $p < 1 \times 10^{-5}$, controlling for age, sex, brain volume, handedness and head motion). Together, these results show that accounting for network strength does not change (or can improve) the results we obtain in the main text. While average network strength does contribute to network controllability measures, it cannot account for our findings, which depend on the particular network topology even given the same average network strength.

Controlling for network density

In addition, in this section we verify that network degree (number of all non-zero edges in the network) does not explain our results, by including network density as a covariate in our calculations. We find that mean average controllability remains strongly correlated with mean modal controllability: Pearson's correlation coefficient $r = 0.88$, $df = 881$, $p < 1 \times 10^{-5}$. These controllability metrics continue to display strong negative correlations with synchronizability: $r = -0.85$, $df = 881$, $p < 1 \times 10^{-5}$ for mean average controllability and $r = -0.85$, $df = 881$, $p < 1 \times 10^{-5}$ for mean modal controllability, respectively.

In examining the relationships between these metrics and age across the sample from ages 8 to 22, we find that our results persist when including network density as an additional covariate (in addition to sex, brain volume, handedness and head motion). Mean average controllability remains positively correlated with age ($r = 0.28$, $df = 881$, $p < 1 \times 10^{-5}$) and mean modal controllability does as well ($r = 0.25$, $df = 881$, $p < 1 \times 10^{-5}$). Synchronizability continues to show a strong negative correlation with age: $r = -0.33$, $df = 881$, $p < 1 \times 10^{-5}$. Taken together, these results indicate that our findings on controllability, synchronizability, and their changes across development are not driven by network density.

Application of streamline thresholds to minimize the probability of spurious edges

To verify that the relationships between controllability or synchronizability with age are not driven by the effect of spurious streamlines in network analysis, we tested

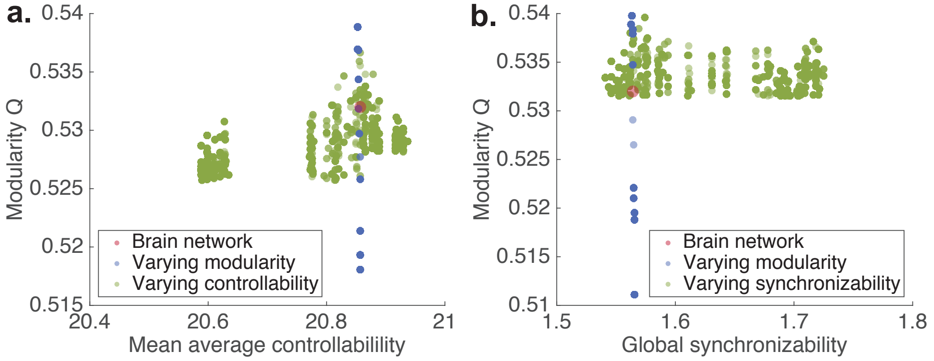

Supplementary Figure 4: **Lack of mathematical dependence between modularity and controllability or synchronizability.** Re-wiring of a brain network (red dot) while keeping the strength distribution constant, in a similar manner to Pareto-optimization, shows that modularity and mean average controllability or synchronizability, can vary independently from each other. **a.** Green dots denote re-wired networks that vary in mean average controllability while their modularity varies little. Blue dots denote re-wired networks that vary in modularity while their mean average controllability varies little. **b.** Green dots denote re-wired networks that vary in global synchronizability while their modularity varies little. Blue dots denote re-wired networks that vary in modularity while their synchronizability varies little.

whether our findings are robust to the application of absolute streamline count thresholds, to minimize the probability of spurious edges. To do this, we apply an absolute cut-off to the streamline counts present in the structural connectivity networks, where edges with fiber counts less than and up to the threshold are set to zero. We use a range of thresholds that change the corresponding network densities and show that when reducing the average network density down to as low as 60% of the original networks, our results remain robust.

We begin by using a threshold of 1 streamline count (i.e. only streamlines of weight 2 and above are retained), which reduces the average network density from 17% to 15%. We find that the mean average controllability remains significantly correlated with age (Pearson's correlation coefficient $r = 0.28$, $df = 881$, $p < 1 \times 10^{-5}$), as does mean modal controllability ($r = 0.22$, $df = 881$, $p < 1 \times 10^{-5}$), while synchronizability remains significantly negatively correlated ($r = -0.37$, $df = 881$, $p < 1 \times 10^{-5}$). All of these computations control for sex, head motion, brain volume and handedness as in the main analyses. We repeat this analysis next using a threshold of 5 streamline counts, which reduces the average network density further to 12%. In this case, all these results remain the same (up to reported precision), i.e. mean average controllability remains as $r = 0.28$, $df = 881$, $p < 1 \times 10^{-5}$, mean modal controllability still gives $r = 0.22$, $df = 881$, $p < 1 \times 10^{-5}$, while synchronizability is still negatively correlated with age: $r = -0.37$, $df = 881$, $p < 1 \times 10^{-5}$.

Lastly, we use a threshold of 10 streamline counts, which brings the average network density down to just 10%. In this case, the results are barely changed, where mean average controllability is still significantly correlated with age ($r = 0.29$, $df = 881$, $p < 1 \times 10^{-5}$), as is mean modal controllability ($r = 0.22$, $df = 881$, $p < 1 \times 10^{-5}$), while synchronizability still shows a negative correlation with age ($r = -0.36$, $df = 881$, $p < 1 \times 10^{-5}$) — all while controlling for sex, handedness, brain volume and head motion. Taken together, our results show that across a range of applied absolute streamline count thresholds, our findings remain robust and hence are not driven by the possible contribution of spurious network edges.

Controlling for modularity

A natural question is whether modularity could be a mediating factor in these results, since one might expect that networks with increasing modular organization show less synchronizability, and therefore may be more controllable. However, we find that the relationship between modularity and controllability or synchronizability is not straightforward, which we demonstrate using a set of numerical experiments.

To show this rigorously, we perform a set of numerical experiments capitalizing on the approaches developed and exercised in the manuscript. Specifically, we conduct a network re-wiring procedure similar to that used in Pareto-optimization, that changes an edge of the network while keeping the overall strength distribution constant. We ask whether it is possible to devise a rewiring scheme that can hold controllability steady while chang-

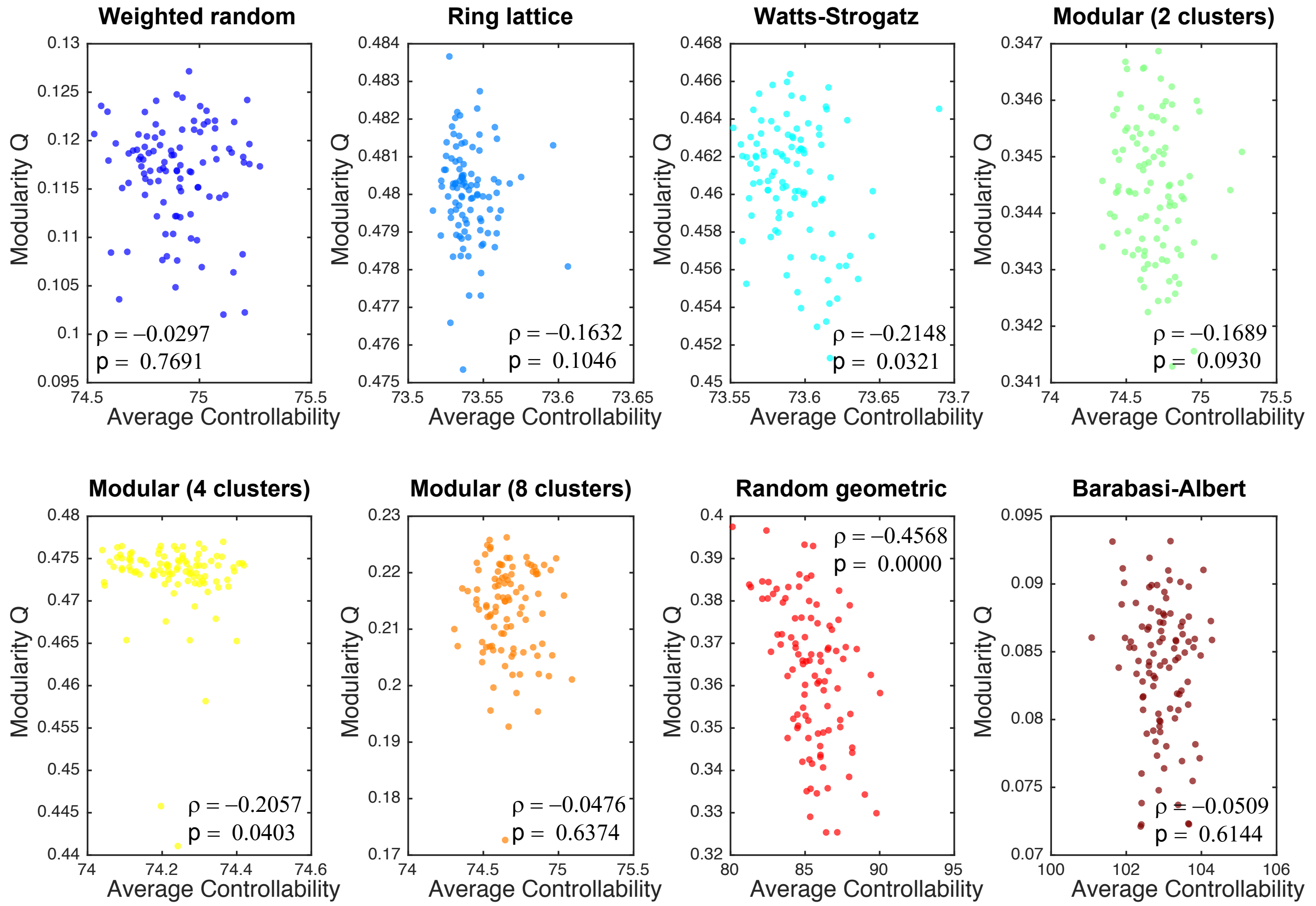

Supplementary Figure 5: **Relation between mean average controllability and modularity Q across graphs for the Gaussian edge weighting scheme.** Mean average controllability and modularity Q values in each graph model ensemble, where scatterplots show values for each graph in the ensemble. Results are shown for the Gaussian edge weighting scheme. The eight graph models include the weighted random graph (WRG), the ring lattice (RL), the Watts-Strogatz small-world (WS), the modular graphs (MD2, MD4, MD8), the random geometric (RG), and the Barabasi-Albert preferential attachment (BA) models.

ing modularity, or hold modularity steady while changing controllability. If it is impossible to devise rewiring schemes to affect these outcomes, then it suggests that modularity and controllability have an inherent mathematical dependence, even if we – as a field – do not yet understand exactly what that mathematical relationship is. On the other hand, if we can successfully change one of these variables while holding the other constant, it suggests that controllability and modularity do not have a strict mathematical dependence on one another.

To enact this numerical experiment, we choose a brain network at random from the ensemble. We rewire the network using Pareto-optimization, at each step calculating the mean average controllability and the modularity as estimated by a maximization of a modularity quality function [1] using a Louvain-like [2] locally greedy algorithm [3] with a structural resolution parameter value of unity [4]. Importantly, we find that the modularity and controllability are not bound together with any clear relationship (Supplementary Fig. 4, left). We also repeat this with synchronizability and modularity, to show that similarly, modularity and synchronizability are not bound together with any clear relationship (Supplementary Fig. 4, right). This numerical experiment demonstrates that controllability and modularity (or synchronizability and modularity) are not mathematically dependent at a fundamental level. More generally, these results suggest that modularity cannot simply explain the observed correlation between controllability and synchronizability as a function of age during normative neurodevelopment.

To further demonstrate that our results are not driven by modularity, we here include the modularity metric Q as a covariate in our calculations. In this case, we run

100 iterations of a Louvain-like locally greedy algorithm to maximize the modularity quality function with $\gamma = 1$ for each subject's structural adjacency matrix [4]. For each subject we then obtain a consensus partition [4] and the consensus Q value. We then calculate the relationship between synchronizability and controllability after regressing out the effects of modularity. We find that mean average controllability remains strongly correlated with mean modal controllability: Pearson's correlation coefficient $r = 0.87$, $df = 881$, $p < 1 \times 10^{-5}$. These controllability metrics continue to display strong negative correlations with synchronizability: $r = -0.84$, $df = 881$, $p < 1 \times 10^{-5}$ for mean average controllability and $r = -0.81$, $df = 881$, $p < 1 \times 10^{-5}$ for mean modal controllability, respectively. These data suggest that individual differences in modularity are not sufficient to explain the relationship between controllability and synchronizability.

Furthermore, in examining the relationships between these metrics and age across the sample of youth from ages 8 to 22, we observe that our findings remain significant when including modularity as one of the covariates, along with sex, brain volume, handedness and head motion. Mean average controllability remains positively correlated with age ($r = 0.28$, $df = 881$, $p < 1 \times 10^{-5}$) as does mean modal controllability ($r = 0.21$, $df = 881$, $p < 1 \times 10^{-5}$). Synchronizability continues to show a strong negative correlation with age: $r = -0.36$, $df = 881$, $p < 1 \times 10^{-5}$. Taken together, these results indicate that our findings on controllability, synchronizability, and their changes across development are not driven by the varying modular structure of the networks.

In addition, modularity and controllability can be computationally related to one another in some classes of graphs, but are not related to one another in other classes of graphs. Hence, there is no instrinsic relationship between these two quantities, as we demonstrate by investigating various families of networks below.

For our ensemble of brain graphs, we find that modularity is negatively correlated with mean average controllability $r = -0.26$, as is mean modal controllability, $r = -0.39$. Synchronizability is actually positively correlated with modularity, $r = 0.37$. We illustrate that different families of graphs will show different relationships between controllability and modularity, by simulating ensembles of networks (100 instantiations each) for 8 varying graph topologies, where each of these sets of 8 x 100 topologies are overlaid with two different edge weight distributions. For a given edge weight distribution – Gaussian (Fig. 5) or streamline counts (Fig. 6) – we find that when holding the network size and edge density constant, the network topology alone gives strongly varying relationships between average controllability and modularity. A summary of the Spearman ρ correlation coefficients and associated p-values for all 2 x 8 graph types is given in Table. 1 in the main text. For more details on graph generation and edge weight assignment, please see the Methods subsection "Network generation".

Collectively, these results indicate that the relationship of modularity with controllability is not consistent across different families of graph types or edge weight distributions, and hence that modularity cannot be considered to be a fundamental driver or proxy for our results on controllability or synchronizability.

Lastly, we also examine the effect of changes in modularity in both a simulated modular network (as generated from the Brain Connectivity Toolbox) and an empirical brain network. This is done by altering the network modularity through random edge swaps while leaving controllability and synchronizability unconstrained, see Fig. 7. We start with a modular small-world network (top row), and perform several iterations of edge swaps, in order to maintain the same network weight distribution but alter its topology. We find that different runs change controllability and synchronizability in inconsistent ways, which depend on the particular edges being changed – and that increasing modularity (blue tones) and decreasing modularity (green tones) both can produce increasing *and* decreasing controllability and synchronizability.

These simulations are repeated starting from an empirical brain network (bottom row), where again we find inconsistent impact on controllability and synchronizability upon altering modularity. Similarly, increasing modularity (blue tones) and decreasing modularity (green tones) both can produce increasing *and* decreasing controllability and synchronizability. Further, any global trends that may be present are very different between the modular small-world network and the brain network, especially in the dependence of controllability on changes in modularity. Taken together, these results demonstrate that our results are not driven by changes in modularity.

Notably, the same modularity index Q can broadly describe many different topologies of networks, which may have different controllability or synchronizability. For instance, we can understand synchronizability as a measure of the variance of length scales within the network (captured by the eigenvalues of the Laplacian matrix). When a modular network becomes more randomized, this can be done in a way that either increases the heterogeneity of length scales in the network, or homogenizes them – depending on the initial topology of the network and the precise re-wiring done during the randomization. More generally, questions regarding the development of mathematical relations between modularity and synchronizability or controllability are extremely fascinating and pose an excellent avenue for further exploration in the future; however such efforts are beyond the scope of our present work.

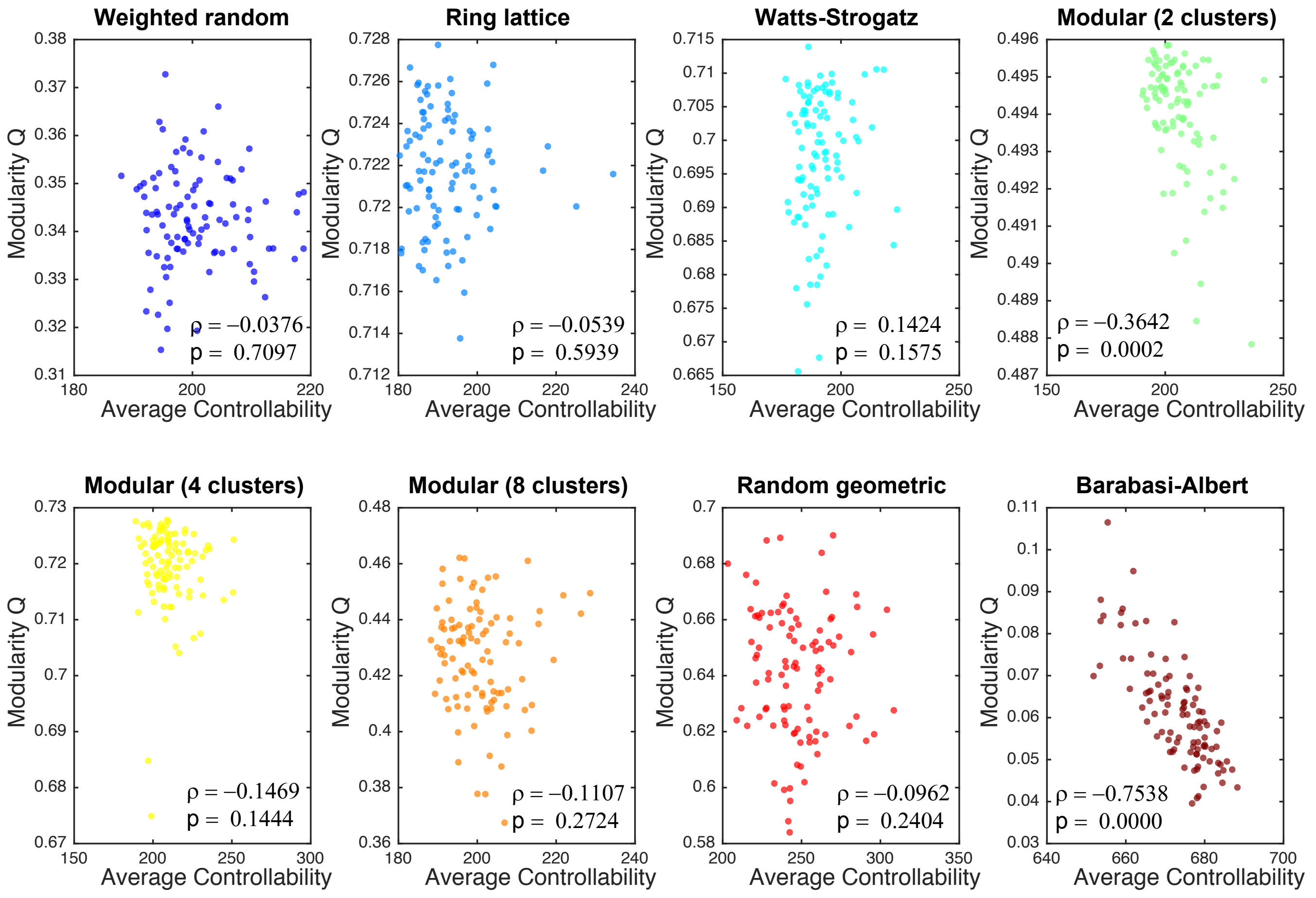

Supplementary Figure 6: **Relation between mean average controllability and modularity Q across graphs for the streamline counts weighting scheme.** Mean average controllability and modularity Q values in each graph model ensemble, where scatterplots show values for each graph in the ensemble. Results are shown for the streamline counts weighting scheme. The eight graph models include the weighted random graph (WRG), the ring lattice (RL), the Watts-Strogatz small-world (WS), the modular graphs (MD2, MD4, MD8), the random geometric (RG), and the Barabasi-Albert preferential attachment (BA) models.

Subjects with lowest in-scanner head motion

Our work employs stringent restrictions to rule out head motion during the scanning procedure as a potential confounding factor, by ensuring that data have passed rigorous visual and automatic quality assurance to detect head motion [5]. We excluded subjects with in-scanner head motion of above 2mm (see Methods) and controlled for motion in all analyses using the 882 subject sample.

As a last check, we verify that the significant results we observe across the entire sample are replicable on the 200 subjects with the lowest head motion. While all 882 subjects have an average head motion of 0.45mm mean relative displacement, here we retain the 200 subjects with the lowest relative head motion (below 0.22mm) to replicate our findings. We find that these subjects with lowest in-scanner head motion still display a positive correlation between whole-brain average and modal controllabilities (Pearson's correlation coefficient $r = 0.86$, $df = 199$, $p < 1 \times 10^{-5}$), while synchronizability remains negatively correlated with both mean average and modal controllabilities ($r = -0.84$, $df = 199$, $p < 1 \times 10^{-5}$ and $r = -0.84$, $df = 199$, $p < 1 \times 10^{-5}$ respectively).

We also find that these subjects display increasing controllability with age, for both mean average controllability (Pearson's correlation coefficient $r = 0.20$, $df = 199$, $p = 5 \times 10^{-3}$) and mean modal controllability ($r = 0.16$, $df = 199$, $p = 4 \times 10^{-2}$). Synchronizability decreases with age: $r = -0.27$, $df = 199$, $p = 1 \times 10^{-4}$; and sex, brain volume, handedness and head motion have been controlled for. These results are consistent with our findings in the main text, although the p-values are larger as expected for this smaller sample size. There is a similar emergence of 'super-controllers' where brain regions with

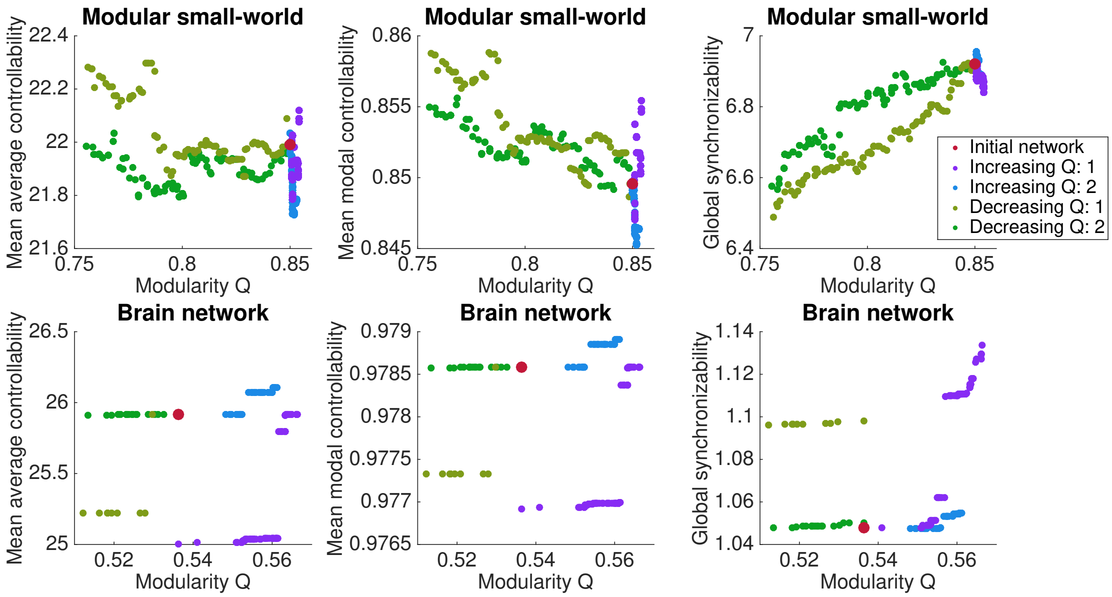

Supplementary Figure 7: **Varying modularity in a modular network and an empirical brain network.** We examine the effect of changes in modularity in both a generated modular network (top) and the empirical brain network of a single subject (bottom). Using edge swaps, network modularity can be altered while controllability (left and middle) and synchronizability (right) are unconstrained. Starting from the same initial network (red dot), this randomization can be repeated, where we show two instances of increasing modularity (blue tones) and two instances of decreasing modularity (green tones) for each network. Different runs change controllability and synchronizability in inconsistent ways, which depend on the particular edges being changed, demonstrating that increasing modularity and decreasing modularity can both produce increasing *and* decreasing controllability and synchronizability. Furthermore, any global trends that may be present are very different between the modular small-world network and brain network, especially in the dependence of controllability on changes in modularity.

higher average and modal controllability, are also increasing in their controllability with age more than regions with low controllability. Regions that display increasing average controllability with age are positively correlated with their average controllability values: $r = 0.60$, $df = 233$, $p < 1 \times 10^{-5}$, just as regions that display increasing modal controllability with age are also positively correlated with their modal controllability values: $r = 0.38$, $df = 233$, $p < 1 \times 10^{-5}$. Lastly, the stable controllers in the subcortical region again show a negative correlation between the mean average controllability in those regions, and the subject's IQ (Spearman correlation coefficient $\rho = -0.19$, $df = 233$, $p = 8 \times 10^{-3}$, controlling for age, sex, brain volume, handedness and head motion). Together, these findings match well with the results we obtain in the main text, and rule out head motion as a confounding factor for our conclusions.

Volume-normalized streamline connectivity

In the main text we use the raw number of streamline counts between brain regions as a measure of connectivity in our networks. Aware that larger regions are likely to have more streamlines that begin and end in them, we normalize each streamline count by the the total volume of the node pair [6]. This results in brain networks with much smaller weights (average strength of 0.011) as compared to the unnormalized networks (average strength of 19). When repeating our analysis on these normalized networks, in order to obtain controllability metrics that can be reasonably compared with those from the unnormalized networks, the internal normalization of $1+\xi_0(\mathbf{A})$ has to be modified accordingly (where $\xi_0(\mathbf{A})$ is the largest singular value of the network adjacency matrix $\mathbf{A}$; see Methods). For consistency of analysis, we choose a new normalization of $f + \xi_0(\mathbf{A})$, where $f = 0.011/19$ — the ratio between the average strengths of the normalized to unnormalized networks respectively.

Here we find that the 190 adults still display a positive correlation between whole-brain average and modal controllabilities (Pearson's correlation coefficient $r = 0.67$, $df = 189$, $p < 1 \times 10^{-5}$), and synchronizability is negatively correlated with both mean average and modal controllabilities ($r = -0.49$, $df = 189$, $p < 1 \times 10^{-5}$ and $r = -0.62$, $df = 189$, $p < 1 \times 10^{-5}$ respectively).

In the entire youth sample from ages 8 to 22, we again see that mean average controllability and mean modal controllability are both positively correlated with age: Pearson's correlation coefficient $r = 0.28$, $df = 881$, $p < 1 \times 10^{-5}$ and $r = 0.24$, $df = 881$, $p < 1 \times 10^{-5}$ respectively. Synchronizability shows an extremely strong negative correlation with age: Pearson's correlation coefficient $r = -0.49$, $df = 881$, $p < 1 \times 10^{-5}$. These results are controlled for sex, brain volume, handedness and head motion, and replicate well our findings for the unnormalized streamline connectivity.

On the regional level, we still see the presence of 'super-controllers' where brain regions with higher average and modal controllability are also increasing in their controllability with age more than the regions with low controllability. Regions that display increasing average controllability with age are positively correlated with their average controllability values: $r = 0.60$, $df = 233$, $p < 1 \times 10^{-5}$, just as regions that display increasing modal controllability with age are also positively correlated with their modal controllability values: $r = 0.48$, $df = 233$, $p < 1 \times 10^{-5}$. Lastly, the stable controllers in the subcortical region again show a negative correlation between the mean average controllability in those regions, and the subject's cognitive performance (Spearman correlation coefficient $\rho = -0.067$, $df = 233$, $p = 5 \times 10^{-2}$, controlling for age, sex, brain volume, handedness and head motion). Together, these findings are consistent with the results we obtain using unnormalized streamlines.

Different parcellation scale

The analysis in the main text relies on brain networks that have been constructed on regions assigned from the Lausanne atlas at the scale of 234 regions [7]. Here we repeat our analysis on networks constructed at a finer scale of 463 brain regions in this atlas. As in the earlier sections, we find that the 190 adults still display a positive correlation between whole-brain average and modal controllabilities (Pearson's correlation coefficient $r = 0.85$, $df = 189$, $p < 1 \times 10^{-5}$), and synchronizability is negatively correlated with both mean average and modal controllabilities ($r = -0.81$, $df = 189$, $p < 1 \times 10^{-5}$ and $r = -0.74$, $df = 189$, $p < 1 \times 10^{-5}$ respectively). In the entire youth sample from ages 8 to 22, we also see that mean average controllability and mean modal controllability are both positively correlated with age: Pearson's correlation coefficient $r = 0.28$, $df = 881$, $p < 1 \times 10^{-5}$ and $r = 0.19$, $df = 881$, $p < 1 \times 10^{-5}$ respectively. Synchronizability also decreases with age: $r = -0.36$, $df = 881$, $p < 1 \times 10^{-5}$, where we control for sex, brain volume, handedness and head motion. These results all replicate our findings at the 234-region scale well.

On the regional level, there is again the presence of 'super-controllers' where brain regions with higher average and modal controllability are also increasing more in their controllability with age. Regions that display increasing average controllability with age are positively correlated with their average controllability values: $r = 0.35$, $df = 233$, $p < 1 \times 10^{-5}$, just as regions that display increasing modal controllability with age are also positively correlated with their modal controllability values: $r = 0.27$, $df = 233$, $p < 1 \times 10^{-5}$. In this parcellation, the stable controllers in the subcortical region show no significant correlation between the mean average controllability in those regions and the subject's cognitive performance (Spearman correlation $p = 0.71$), after controlling for age, sex, brain volume, handedness and head motion, suggesting that the finer parcellation masks the global drivers of individual differences in cognitive performance.

Probabilistic tractography

We used a ball and two sticks multi-compartment fiber model to fit the DTI data using the FSL bedpostx algorithm [8], which utilizes Markov chain Monte Carlo sampling to estimate uncertainty of fiber orientations at each voxel. For probabilistic tractography, we generated subject-specific seed volumes at the intersection of 234 dilated gray matter regions and the FreeSurfer GM-WM boundary [9]. We ran FSL probtrackx [10], initiating 1000 probabilistic samples in each GM-WM boundary voxel identified in the 234 seed regions. Otherwise, we used default tracking parameters (a step-length of 0.5mm, 2000 steps maximum, curvature threshold of 0.02). We retained 878 subjects from the original sample, which passed the quality control measures for probabilistic tractography.

Networks were constructed where edge weights were equal to the number of probabilistic streamlines connecting each region pair [11]. As the resulting matrices have many more weak connections, thresholding of the lowest weights was done to obtain matrices of equivalent density (17%) to the deterministically obtained matrices. Normalization for each subject by their total network strength was also performed, and for simplicity we report the statistics for the whole youth sample. We find that mean average controllability and mean modal controllability remain positively correlated (Pearson's correlation coefficient $r = 0.83$, $df = 877$, $p < 1 \times 10^{-5}$), and remain both negatively correlated with global synchronizability ($r = -0.90$, $df = 877$, $p < 1 \times 10^{-5}$ and $r = -0.83$, $df = 877$, $p < 1 \times 10^{-5}$, respectively). The trends with age remain robust: mean average controllability increases with age (Pearson's correlation coefficient $r = 0.33$, $df = 877$, $p < 1 \times 10^{-5}$) as does mean modal controllability ($r = 0.25$, $df = 877$, $p < 1 \times 10^{-5}$). Synchronizability decreases with age: $r = -0.32$, $df = 877$, $p < 1 \times 10^{-5}$, where we control for sex, brain volume,

handedness and head motion.

The above results demonstrate consistency with findings obtained from determinstic tractography. Still, it will be important in future to determine a data-driven method for which to threshold the probabilistic tractography matrix, and better understand the role of these weak connections in controllability and synchronizability.

SUPPLEMENTARY REFERENCES

[1] M. E. J. Newman, Proc. Natl. Acad. Sci. U.S.A. **103**, 8577 (2006).

[2] V. D. Blondel, J.-L. Guillaume, R. Lambiotte, and E. Lefebvre, Journal of Statistical Mechanics: Theory and Experiment **2008** (2008).

[3] P. J. Mucha, T. Richardson, K. Macon, M. A. Porter, and J.-P. Onnela, Science **328**, 876 (2010).

[4] D. S. Bassett, M. A. Porter, N. F. Wymbs, S. T. Grafton, J. M. Carlson, and P. J. Mucha, Chaos **23**, 013142 (2013).

[5] D. R. Roalf, M. Quarmley, M. A. Elliott, T. D. Satterthwaite, S. N. Vandekar, K. Ruparel, E. D. Gennatas, M. E. Calkins, T. M. Moore, R. Hopson, K. Prabhakaran, C. T. Jackson, R. Verma, H. Hakonarson, R. C. Gur, and R. E. Gur, NeuroImage **125**, 903 (2016).

[6] P. Hagmann, L. Cammoun, X. Gigandet, R. Meuli, C. J. Honey, V. J. Wedeen, and O. Sporns, PLoS Biol **6**, 1 (2008).

[7] A. Daducci, S. Gerhard, A. Griffa, A. Lemkaddem, L. Cammoun, X. Gigandet, R. Meuli, P. Hagmann, and J.-P. Thiran, PLoS ONE **7**, 1 (2012).

[8] T. E. J. Behrens, H. Johansen-Berg, M. W. Woolrich, S. M. Smith, C. A. M. Wheeler-Kingshott, P. A. Boulby, G. J. Barker, E. L. Sillery, K. Sheehan, O. Ciccarelli, A. J. Thompson, J. M. Brady, and P. M. Matthews, Nat Neurosci **6**, 750 (2003).

[9] L. Li, J. K. Rilling, T. M. Preuss, M. F. Glasser, and X. Hu, Human Brain Mapping **33**, 1894 (2012).

[10] T. Behrens, H. J. Berg, S. Jbabdi, M. Rushworth, and M. Woolrich, NeuroImage **34**, 144 (2007).

[11] J. M. Duarte-Carvajalino, N. Jahanshad, C. Lenglet, K. L. McMahon, G. I. de Zubicaray, N. G. Martin, M. J. Wright, P. M. Thompson, and G. Sapiro, Neuroimage **59**, 3784 (2012).
